# An Anthocyanin-Based Eco-Friendly Triboelectric Nanogenerator for pH Monitoring and Energy Harvesting

**DOI:** 10.3390/molecules29091925

**Published:** 2024-04-23

**Authors:** Wuliang Sun, Junhui Dong, Wenbo Li, Xiaobo Gao, Jun Liu, Ding Nan

**Affiliations:** 1 School of Materials Science and Engineering, Inner Mongolia University of Technology, Hohhot 010051, China; 2 College of Chemistry and Chemical Engineering, Inner Mongolia University, Hohhot 010021, China; 3 College of Food Science and Engineering, Inner Mongolia Agricultural University, Hohhot 010018, China; 4 Institute of Applied Nanotechnology, Jiaxing 314031, China; 5 Beijing Institute of Nanoenergy and Nanosystems, Chinese Academy of Sciences, Beijing 101400, China

**Keywords:** environmentally friendly, triboelectric nanogenerator, pH monitoring, energy harvesting

## Abstract

In recent years, renewable and sustainable triboelectric nanogenerators have attracted attention due to their high energy conversion rate, and enhancing their functionality further contributes to their applicability across various fields. A pH-sensitive triboelectric nanogenerator (pH-TENG) has been prepared by electrostatic spinning technology, with anthocyanin as the pH indicator and environmentally friendly polyvinyl alcohol (PVA) as the substrate. Among many friction-negative materials, the pH-TENG exhibits the best combination with fluorinated ethylene propylene (FEP) and yields an open-circuit voltage of 62 V, a short-circuit current of 370 nA, and a transferred charge of 21.8 nC. At a frequency of 3 Hz, it can charge a 4.7 μF capacitor to 2 V within 45 s, effectively powering a thermometer. Furthermore, the presence of anthocyanin does not affect the pH-TENG’s power generation performance and enables the monitoring of a wide range of environmental pH changes, with an ΔE change of 28.8 ± 7.6. Therefore, pH-TENG prepared with environmentally friendly materials can bring new available materials to the biological and medical fields.

## 1. Introduction

In today’s world, the rising electricity demand driven by smart devices, population growth, and economic activities intensifies the focus on renewable, sustainable, and distributed power sources [1]. However, it is worth noting that traditional energy resources like fossil fuels, our primary energy sources for survival, face challenges related to depletion and environmental pollution. Simultaneously, while batteries remain a reliable portable power source, they come with limitations, such as a finite lifespan and environmental concerns associated with maintenance and disposal [2,3]. Consequently, searching for renewable energy sources has become an imperative part of human development. One of the most promising solutions in this quest is the design of devices capable of harnessing energy from the surrounding environment. Researchers have also actively explored various harvesting technologies for environmental energy (solar and bioenergy) [4,5]. These encompass a wide range of sources, from the low-intensity kinetic energy generated by random movements and human activities (such as walking, running, jumping, and breathing) to mechanical vibrations produced by machinery and the fluid flow in wind, oceans, and rivers [6,7,8,9,10]. Among the environmental energy-harvesting technologies, the triboelectric nanogenerator (TENG), invented by Dr. Zhong Lin Wang in 2012, has attracted significant attention [11,12,13]. TENG stands out due to its exceptional energy conversion efficiency, straightforward structure, and robust scalability. Consequently, it has emerged as a highly promising energy-harvesting technology, particularly in nanoscale energy systems [14]. 

The TENG is a device that harnesses the triboelectric effect and electrostatic induction to convert mechanical energy into electrical energy. Currently, it encompasses four distinct operational modes: vertical contact-separation mode, single-electrode mode, lateral-sliding mode, and freestanding triboelectric-layer mode [15]. These four working modes are universally applicable for energy harvesting and self-powered sensing in daily life [16,17]. Based on these four operational modes, TENG can be further developed into functional TENGs with a variety of properties, such as flame retardancy, antibacterial activity, self-cleaning capabilities, biomimicry, sensing, environmental adaptability, and green environmental protection [18,19,20]. Among these, green environmentally friendly TENGs have been widely researched due to their green, low-cost, and sustainable energy-harvesting and sensing capabilities. The key factor for green TENGs lies in the choice of materials. Some researchers have approached it from an environmental and low-cost direction, using natural materials such as wood, flowers, gelatin-based biodegradable materials, and cellulose materials to fabricate environmentally friendly TENGs [20,21,22,23]. These TENGs can achieve energy harvesting and various applications of self-powered sensors without harming the environment.

Since its introduction in 1934, electrospinning has emerged as a promising technology for producing multifunctional nanofibers [24]. It offers a versatile platform for transforming viscous polymers into films, threads, and fiber mats under a high-voltage electric field [25]. Through this process, nanofibers with small dimensions and high surface-to-volume ratios can be produced, resulting in good performance of the film, such as high sensitivity [26]. Consequently, electrospinning has diverse applications in electronics, biology, energy storage, healthcare, and textile technologies [27]. In 2014, electrospinning debuted in triboelectric nanogenerators (TENGs) [28]. Since that milestone, nanofibers fabricated from a range of polymers, including polyvinylidene fluoride (PVDF), polylactic acid (PLA), nylon, and others, have gained widespread adoption as either positive or negative friction layers within TENGs, and the fibrous structure of these nanofibers augments the electrical properties of polymer materials [29]. Furthermore, nanofiber films, due to their pliable, breathable, and thin nature, serve as versatile sensors for monitoring various human physiological activities (respiration, running, walking, and joint bending), as well as detecting changes in chemical information in the surrounding environment (alterations in temperature, humidity, and ammonia levels) [30,31]. However, it is important to note that most reported TENGs are manufactured using synthetic polymers, metals, metal oxides, and similar materials, often lacking the required biocompatibility [32,33]. This constrains their potential applications within the biomedical, wearable, and implantable realms. Consequently, there has been substantial interest in selecting naturally biodegradable and environmentally friendly polymers for TENG fabrication.

Polyvinyl alcohol (PVA) was first discovered in 1924 through the saponification of poly (vinyl ester) with caustic soda solution. It is a biodegradable synthetic polymer renowned for its exceptional properties, including low cost, biocompatibility, biodegradability, chemical resistance, strong adhesion, non-toxicity, and high mechanical strength [34]. These outstanding properties establish PVA as an ideal environmentally friendly material used in various applications, including paper, medical materials, packaging, and agricultural films [35].

The pH indicator consists of two parts: a support and a pH-responsive dye. Indicators are prepared in a variety of ways (adsorption, covalent bonding, polymer-based matrix, and dyes) [36]. Various synthetic dyes such as Methyl Red, Phenol Red, Bromocresol Green, Bromothymol Blue, Chlorophenol Red, Bromophenol Blue, and Dimethyl Yellow have been utilized in the preparation of pH-responsive chromic indicators. However, most synthetic dyes are toxic, and some even possess carcinogenic properties, which may pose significant threats to life and the environment [37,38]. Recently, the emergence of various natural pigments has addressed this issue. Substances such as anthocyanins, curcumin, alizarin, betaine, and green tea extracts have been employed as pH indicator dyes [38,39,40,41]. Among them, anthocyanins are the most extensively used due to their functional capacity to change color over a wide pH range, their safety, and their plentiful availability.

Herein, a pH-sensitive triboelectric nanogenerator (pH-TENG) was developed to monitor environmental pH and efficiently harvest mechanical energy. The pH-TENG is dominated by a PVA@Ac nanofiber film, prepared by the electrostatic spinning technique and the chemical crosslinking method with PVA as the substrate and anthocyanin as the indicator material. FEP is employed as the friction layer to assist the single-electrode pH-TENG in collecting low-intensity mechanical energy from the environment, generating a maximum output of 40.8 μW. Furthermore, the presence of anthocyanin enables the pH-TENG to visually and in real-time monitor the environmental pH. This pH-TENG, composed of environmentally friendly materials, brings new available materials to the biological and medical fields.

## 2. Results and Discussion

**Figure 1:**
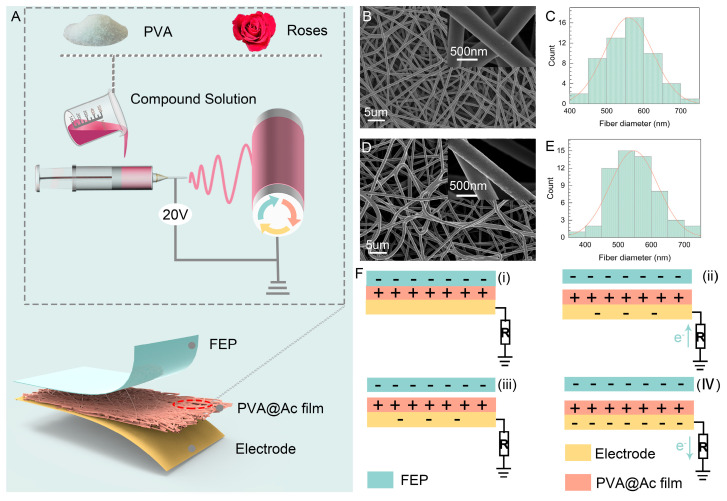
(**A**) Preparation process of pH-TENG. (**B**) SEM image of crosslinked PVA fiber film. (**C**) Fiber diameter distribution of crosslinked PVA fiber film. (**D**) SEM image of PVA@Ac fiber film. (**E**) Fiber diameter distribution of PVA@Ac fiber film. (**F**) Schematic illustrating the operational mechanism of the pH-TENG in a single-electrode mode (i–iv are the four processes for TENG to generate an electrical signal).

Herein, we propose a simple and easily scalable method for preparing pH-sensitive triboelectric nanogenerators (pH-TENGs). As shown in Figure 1A, the pH-TENG was prepared through the electrostatic spinning of a mixture of PVA and anthocyanin. PVA was dissolved in water to obtain a clear and viscous solution. Then, rose anthocyanin extract was added and stirred to obtain a red composite solution. Under the high-voltage electric, the mixed solution was transformed into the nanofiber film. Subsequently, to prepare pH-TENG with superior performance, the fiber films were chemically crosslinked to enhance their water resistance, pH sensitivity, and environmental friendliness. This process is mainly a reaction between PVA and glutaraldehyde (GA), including intramolecular and/or intermolecular crosslinking. Specifically, there is a condensation reaction between the hydroxyl groups in PVA and the difunctional aldehyde molecules in GA to form an aldehyde bridge (as shown in Figure S1) [42]. Within the 3075–3660 cm^−1^ range, a peak corresponding to the vibrational modes of hydroxyl groups in intramolecular and intermolecular hydrogen bonds was observed. The peak intensity of crosslinked PVA was lower than that of pure PVA, which can be attributed to the reaction between some hydroxyl groups of pure PVA and glutaraldehyde, thus furnishing the direct evidence of crosslinking within the PVA (Figure S2) [43]. As illustrated in Figure 1B,C and Supplementary Figure S3A, the SEM observations demonstrate that nanofiber films derived from various treatment groups, including pure PVA, crosslinked PVA, and PVA@Ac, uniformly exhibit the formation of continuous and smooth nanofibers. The nanofiber diameters were analyzed using Image Pro 6, and the results, depicted in Figure 1D,E, as well as Supplementary Figure S3B, indicate that neither the crosslinking process (558 nm for crosslinked PVA) nor the incorporation of anthocyanin (553 nm for PVA@Ac) significantly alters the diameter variations.

The working principal schematic of the pH-TENG is shown in Figure 1F. The pH-TENG’s fiber film operates in a single-electrode TENG mode, whose underlying principle is transferring charges resulting from contact and separation. In this process, the PVA@Ac film loses electrons more readily than the FEP layer, becoming the positive electrode, whereas the FEP becomes the negative electrode. When the PVA@Ac film comes into contact with FEP due to the applied force, an electrochemical interaction occurs at the interface, generating an equivalent number of polar charges. The charges are in equilibrium at this stage, resulting in the absence of an electric current (Figure 1F(i)). However, once the force is removed and the PVA@Ac film separates from the FEP, the positive charges induced on the surface of the PVA@Ac film prompt the electrodes to generate negative charges, causing free electrons to move toward the electrodes and generating an electric current (Figure 1F(ii)). Conversely, in the opposite scenario, when the PVA@Ac film approaches the FEP, free electrons flow back to the ground to offset the potential difference, generating a reverse current signal (Figure 1F(iv)).

**Figure 2:**
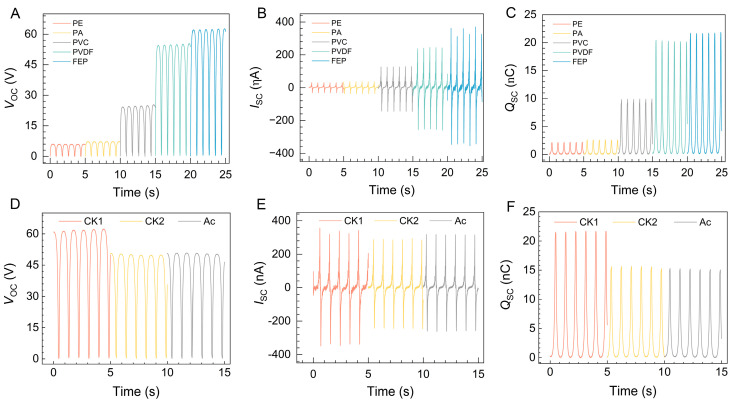
(**A**–**C**) Effect of friction layers on the electrical output performance of the pH-TENG with *V_OC_*, *I_SC_*, and *Q_SC_*, respectively. (**D**–**F**) Electrical output capabilities of PVA fiber films, *V_OC_*, *I_SC_*, and *Q_SC_*, respectively. CK1 refers to pure PVA fiber films, CK2 refers to crosslinked PVA fiber films, and Ac refers to PVA@Ac.

Frictional material plays a pivotal role in influencing the output performance of a triboelectric nanogenerator (TENG) [44,45]. Hence, various materials were assessed as contact materials to evaluate the output performance of the pH-TENG and to identify the most suitable contact material. The output performance of different materials (PE—polyethylene; PA—polyamide; PVC—polyvinyl chloride; PVDF—polyvinylidene fluoride; and FEP—fluorinated ethylene propylene) in terms of open-circuit voltage (*V_OC_*), short-circuit current (*I_SC_*), and transferred charge (*Q_SC_*) is illustrated in Figure 2A–C. The results indicate that FEP exhibits higher output performance (*V_OC_* = 62 V, *I_SC_* = 370 nA, *Q_SC_* = 21.8 nC), whereas PE demonstrates the lowest output performance (*V_OC_* = 5.9 V, *I_SC_* = 30.1 nA, *Q_SC_* = 2.1 nC). This variance is attributed to the diverse surface electron affinity of the materials, with the PVA@Ac film being tribopositive and having a higher affinity for the tribonegative material FEP [46]. In contrast, a tribopositive material like PE lacks higher affinity. Consequently, the FEP film was selected as the contact material for the PVA@Ac fiber film.

We also evaluated the power generation performance of different fiber films, as shown in Figure 2D–F. The results indicate that the uncrosslinked pure PVA fiber film exhibited the highest power generation performance (*V_OC_* = 62.3 V, *I_SC_* = 338.7 nA, *Q_SC_* = 21.7 nC). However, the power generation performance of the crosslinked fiber film (V*_OC_* = 49.8 V, *I_SC_* = 287.1 nA, *Q_SC_* = 15.6 nC) exhibited a significant decrease, attributed to the PVA fiber film becoming smoother under the crosslinking vapor. When 2.5% anthocyanins were added, the power generation performance of the fiber film (*V_OC_* = 50.6 V, *I_SC_* = 317.7 nA, *Q_SC_* = 15.1 nC) was found to be similar to that of the crosslinked film, indicating that the addition of anthocyanins does not significantly impact the power generation performance of the pH-TENG.

**Figure 3:**
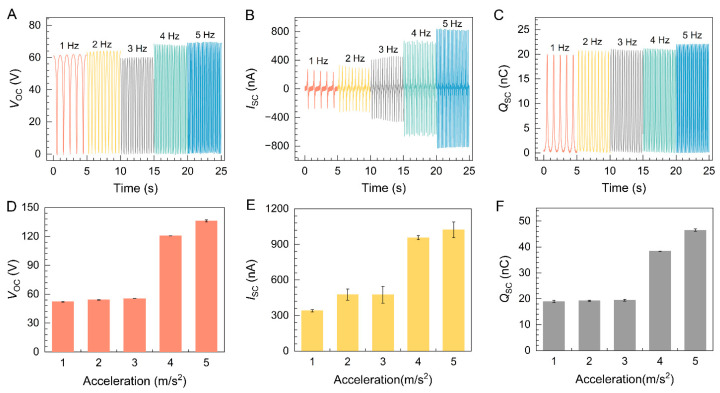
(**A**–**C**) Effect of the loading frequencies (1–5 Hz) on the electrical output performance of the pH-TENG, including *V_OC_*, *I_SC_*, and *Q_SC_*. (**D**–**F**) Effect of the acceleration on the electrical output performance of the pH-TENG, including *V_OC_*, *I_SC_*, and *Q_SC_*.

Due to the irregular and varying nature of mechanical energy from the environment, it is essential to investigate the relationship between the output of the pH-TENG and its frequency [47]. The influence of different frequencies on the power generation performance was examined, and the results are presented in Figure 3A–C. As the frequency increases, the open-circuit voltage remains nearly constant. This behavior can be attributed to the fact that the voltage magnitude primarily depends on the charge density and the separation distance between the plates over a certain period. However, there is an increasing trend in short-circuit current, which is a consequence of the reduced contact time, resulting in an increase in current for the same charge quantity [48]. Furthermore, it was observed that the total transferred charge remains constant at different frequencies.

The impact of transient contact acceleration on pH-TENG’s *V_OC_*, *I_SC_*, and *Q_SC_* was investigated using accelerations ranging from 1 m/s^2^ to 5 m/s^2^. As depicted in Figure 3D–F, *V_OC_*, *I_SC_*, and *Q_SC_* of the pH-TENG increase with the acceleration’s rise, reaching their peak values at 5 m/s^2^ (*V_OC_* = 136.1 V, *I_SC_* = 1021.6 nA, and *Q_SC_* = 46.5 nC). This phenomenon can be attributed to the growing surface contact area of the film during the contact motion, driven by the acceleration increment, thus enhancing the frictional power generation performance. Acceleration plays a critical role in the performance of triboelectric nanogenerators, with the force components within the acceleration serving as key determinants in the process. As acceleration increases, the inertial forces between the FEP and the PVA@Ac nanofiber membrane are amplified, consequently enlarging the contact surface area, which ultimately enhances the electricity-generating performance. Consequently, higher performance can be achieved at higher acceleration during energy harvesting.

**Figure 4:**
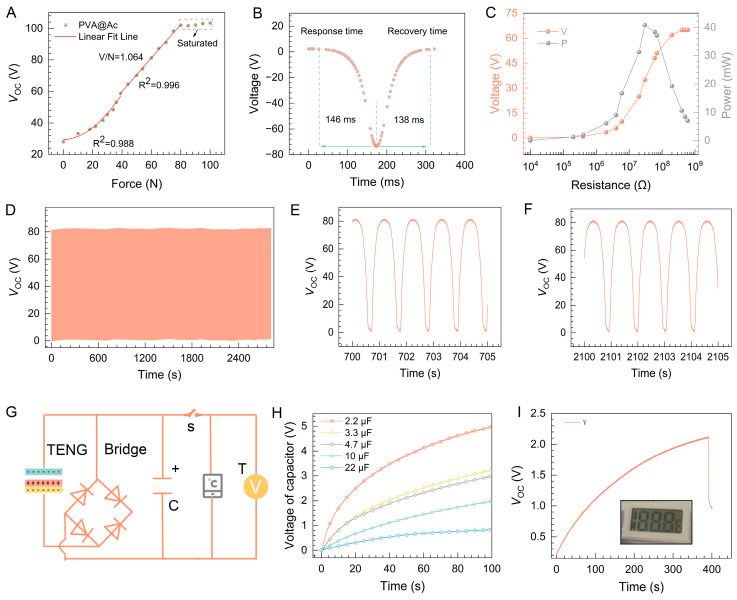
pH-TENG’s performance analysis. (**A**) Correlation and fitting results of pH-TENG output voltage with applied pressure. (**B**) Response time and recovery time of the pH-TENG. (**C**) Current and peak power characteristics of pH-TENG under varied external load resistances. (**D**) Mechanical durability assessment of pH-TENG over 3000 consecutive duty cycles and localized enlargements. (**E**–**G**) The circuit schematic diagram. (**H**) Charging profiles of pH-TENG for different capacitances. (**I**) Voltage profiles of the thermometer during charging and discharging processes.

To ensure a consistent output performance, a linear motor was utilized to apply pressure, with a pressure tester affixed at the contact point of the motor. While monitoring voltage signals and applied pressure, the pressure tester’s probe made direct contact with the pH-TENG. The curve in Figure 4A illustrates the relationship between the output voltage and the varying levels of applied pressure. At the initial pressure setting, the recorded voltage output was 28 V. Subsequent increases in external force yielded a gradual rise in output voltage, transitioning into a linear progression, culminating in a steady-state condition. As external force was incrementally augmented from 0 N to 100 N, the pH-TENG’s output voltage similarly ascended, surging from 28 V to 103 V before plateauing. This phenomenon can be attributed to the amplification of contact quality and the expansion of the effective contact surface area between the two contact layers as external force intensifies, subsequently augmenting the electrical output performance [49].

We also tested its response time, and as shown in Figure 4B, the pH-TENG exhibits excellent pressure sensing sensitivity and accuracy (response time = 146 ms, recovery time = 138 ms). To assess the pH-TENG’s effective output capacity, output voltage measurements were taken under various resistive loads (ranging from 10 KΩ to 1 GΩ). The correlation between output voltage/power and external resistance is depicted in Figure 4C. Output power can be determined using the formula W = U^2^/R, where U represents the output voltage across the external resistance, and R signifies the load resistance. Notably, at a specific load resistance of 30 MΩ, the pH-TENG demonstrated its potential by achieving a maximum output power of 40.8 μW, underscoring its viability as a robust power source. 

Moreover, stability assessments were conducted, as visually depicted in Figure 4D–F. Following an exhaustive 3000 operational cycles, the output voltage maintained remarkable stability in both its waveform and amplitude, affirming the pH-TENG’s commendable stability performance. Following multiple contact-separation cycles, the fibrous membrane continues to maintain its intact surface morphology and fiber structure, as illustrated in Figure S4. During the evaluation of the durability of the pH-TENG, we concurrently assessed the repeatability of its pH-sensing capability. As illustrated in Figure S5, the membrane exhibited consistent chromatic response even after five cycles of exposure to acidic and alkaline conditions. This consistency confirms that the membrane is capable of being recycled for multiple uses and is reliable for the successive measurements of environmental pH fluctuations. For an in-depth evaluation of the pH-TENG’s charging capabilities, as exhibited in Figure 4G,H, a charging regimen was conducted on various capacitors (2.2 μf, 3.3 μf, 4.7 μf, 10 μf, 22 μf) through linear electrodes, with a consistent frequency of 3 Hz. The resultant charging curves of these capacitors within a brief 45 s timeframe revealed the pH-TENG’s ability to rapidly charge a 4.7 μf capacitor to 2 V. This ability highlights the pH-TENG’s aptitude for harnessing low-frequency mechanical energy, rendering it suitable for powering small-scale devices. As showcased in Figure 4I, the pH-TENG’s output voltage was efficiently harnessed, facilitated by a rectifier bridge, to charge a 22 μf capacitor. Subsequently, this charged capacitor could competently supply power to diminutive, low-consumption devices. Figure 4B further underscores this capability, as it demonstrates the pH-TENG’s capacity to charge a capacitor to 2.1 V within a modest 400 s interval, unequivocally affirming its potential to effectively drive a thermometer.

**Figure 5:**
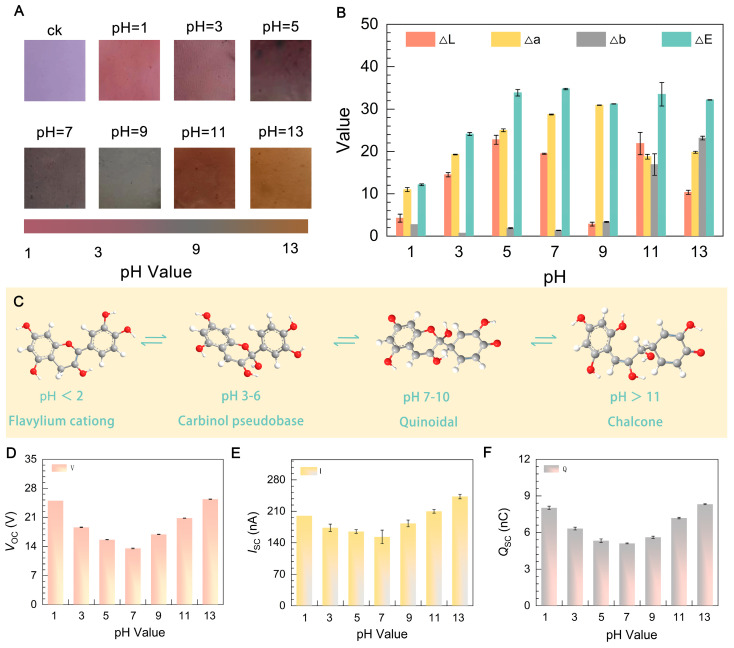
pH-TENG performance and anthocyanin response to pH changes. (**A**) Color variation of pH-TENG at different pH levels. (**B**) Colorimetric values (L*, a*, b*) of pH-TENG at different pH levels. (**C**) Structural transformations of anthocyanin in response to pH variations. (**D**–**F**) Influence of pH on pH-TENG electronic output performance: *V_OC_*, *I_SC_*, and *Q_SC_*.

Polyvinyl alcohol (PVA) is generally considered to be pH-insensitive or pH-neutral (Figure S6, Video S1). However, it is possible to modify PVA to introduce pH sensitivity. One common approach is to incorporate pH-sensitive dyes or indicators into the PVA matrix. Anthocyanin is the most crucial pigment in vascular plants, contributing to the observed red, orange, blue, pink, and purple hues in many plant parts such as flowers, berries, leaves, stems, and roots [50]. The fiber film is endowed with pH sensitivity due to the presence of anthocyanins. As shown in Figure 5A, when exposed to pH 1, the pH-TENG exhibits a deep red color (the flavylium cation). Subsequently, at pH 3, the color lightens (the carbinol pseudobase). Between pH 7 and 9, it assumes a deep gray shade (quinonoidal). As the pH value increases to 13, it transitions to yellow (chalcone). We analyzed the color variation of pH-TENG at different pH levels, as depicted in Figure 5B, and observed an average ΔE change of 28.8 ± 7.6 throughout the experiment. This is primarily determined by changes in L*, a*, and b* values (ΔL = 21.9 ± 6.3, Δa = 13.7 ± 7.6, Δb = 7.1 ± 8.4). The color transition of the film is most pronounced in the red–green shift, followed by white–black, and finally yellow–blue, indicating the pH-TENG’s ability to effectively monitor pH in the environment. Figure 5C and Figure S7 illustrates the structural changes during the anthocyanin color transformation process.

Furthermore, we conducted performance tests on pH-TENG treated with solutions of varying pH levels. Figure 5D–F illustrates the outcomes. In the case of samples treated with acidic solutions at pH = 1, the voltage measured was 22.4 V, the current was 200 nA, and the charge was 7.3 nC. As the pH increased, the electrical performance declined but remained stable. When the pH reached 11, the electrical performance started to rise again, reaching its peak at pH 13, with values of *V_OC_* = 25.4 V, *I_SC_* = 273.3 nA, and *Q_SC_* = 8.1 nC. This indicates that pH-TENG’s power generation performance is influenced by the acidity or alkalinity of the solution. The emergence of these results can be attributed to the acidic/alkaline solutions, causing an increase in the concentration of H^+^ or OH^−^ on the surface of the fibrous membrane, thereby correspondingly augmenting the triboelectric charge density of the pH-TENG. The triboelectric performance exhibits a positive correlation with the rise in ionic concentration, yet it remains independent of the ionic charge polarity. Similar results have been reported by Zhang et al. [51]. This effect may be attributed to the strong acid or alkali conditions causing PVA degradation, resulting in more mechano radicals and enhanced electron transfer. It is also possible that anthocyanin exists in anionic form under acidic and alkaline conditions, further strengthening the inductive effect. The experiment demonstrated that the pH-TENG retains its power generation capabilities after pH detection.

## 3. Materials and Methods

### 3.1. Materials

PVA (88% alcoholysis, Mn = 80,000) was obtained from Aladdin Biochemical Technology Co., Ltd. (Shanghai, China). Anthocyanin was obtained from the extract of rose flowers. Glutaraldehyde, sodium hydroxide, hydrochloric acid, potassium sulfide, potassium hydrogen phthalate, and potassium dihydrogen phosphate were obtained from Sigma-Aldrich (Saint Louis, MO, USA).

### 3.2. Preparation of pH-TENG

Polyvinyl alcohol (12% *w*/*v*) was dissolved in distilled water and anthocyanin (2.5% *w*/*v* rose extract) to prepare a composite polymer solution with stirring at room temperature. The prepared polymer solution was loaded into a 10 mL syringe with a stainless steel needle, and the syringe was mounted in an infusion pump connected to a high-voltage power supply. The electrospinning process was conducted under 20 kV voltage, 0.5 mL/h flow rate, and a collection distance of 16 cm to prepare the fiber film.

The prepared fiber film was fixed on a plastic plate to prevent shrinkage and deformation during the crosslinking. Crosslinking was conducted in a sealed environment using glutaraldehyde and hydrochloric acid vapor. After, the obtained nanofiber film was dried. Finally, pure PVA fiber films (pure PVA), crosslinked PVA fiber films (crosslinked PVA), and crosslinked PVA fiber films with added anthocyanin (PVA@Ac) were established as the subjects of preliminary experimental studies.

The pH-TENG was constructed by sequentially assembling FEP, PVA@AC, and copper electrodes.

### 3.3. Scanning Electron Microscopy (SEM)

After gold coating the various fiber films, the morphology of the nanofiber surfaces was observed using a scanning electron microscope (Quanta FEG 650 form Thermo Fisher Scientific, Waltham, MA, USA), and the diameter of the fibers was determined by selecting randomly 50 fibers using the image processing software Image Plus Pro 6 (Media Cybernetics Co., Ltd. Rockville, MD, USA).

### 3.4. pH Sensitivity Test

To assess the sensitivity of the PVA@AC in the pH-TENG to pH, a series of buffer solutions with pH values ranging from 1 to 13 were prepared at room temperature, conforming to the GB/T604 standard [52], utilizing ultrapure water as the solvent. The PVA@AC was cut into small pieces and put in the prepared pH buffer solutions (pH 1~13). Following complete submersion, the small pieces were immediately removed and placed on filter paper. After 10 min, photographs were taken to document color changes. Additionally, a colorimeter (CR-10 Plus Konica Minolta) was employed to measure the color values of the films. The total color difference (ΔE) was calculated according to Equation (1).

(1) $$\Delta E=\sqrt{(L^{*}-{L^{*}}_{0}{)}^{2}+(a^{*}-{a^{*}}_{0}{)}^{2}+(b^{*}-{b^{*}}_{0}{)}^{2}}$$

L*_0_, a*_0_, and b*_0_ were the initial chromaticity values of the films, and L*, a*, and b* were the color values after the reaction. 

## 4. Conclusions

The single-electrode pH-TENG fabricated using the environmentally friendly material PVA has demonstrated effective operation under low-intensity mechanical energy. To optimize the power generation performance of the pH-TENG, FEP with the highest affinity and best power-generating performance among various frictional negative materials was selected. Under matched impedance conditions, the pH-TENG has an output power of 40.8 μW and can power a thermometer. Additionally, it can monitor changes in environmental pH (1~13) due to the pH sensitivity of anthocyanin. Therefore, this pH-triboelectric nanogenerator, owing to its eco-friendliness, cost-effectiveness, and environmental compatibility, provides a valuable reference for emerging applications in biology and medicine.

## Data Availability

Data are contained within the article.

## Supplementary Materials

The following supporting information can be downloaded at: https://www.mdpi.com/article/10.3390/molecules29091925/s1, Figure S1: (A) SEM image of pure PVA fiber film. (B) Fiber diameter distribution of pure PVA fiber film. Figure S2. FT-IR spectra of different nanofiber membranes. Figure S3. (A) SEM image of pure PVA fiber film. (B) Fiber diameter distribution of pure PVA fiber film. Figure S4. (A) Photographs of PVA@Ac fiber surfaces following the durability experiments. (B) SEM images of PVA@Ac fibers before the durability experiments. (C) SEM images of PVA@Ac fibers after durability testing. Figure S5. The reusability of pH-TENG. Figure S6. The color variations of pure PVA under different pH values. Figure S7. Structural transformations of anthocyanin in response to pH variations. Video S1-The color variations of pure PVA under different pH values.
