# Supplementary material for: An Anthocyanin-Based Eco-Friendly Triboelectric Nanogenerator for pH Monitoring and Energy Harvesting
Source: Molecules. 2024 Apr 23;29(9):1925. doi: 10.3390/molecules29091925 (PMC11085175; doi:10.3390/molecules29091925)
Supplement: Supplementary file 1 [file molecules-29-01925-s001.zip › molecules-2947910-supplementary.pdf]

An Anthocyanin-Based Eco-Friendly Triboelectric Nanogenerator for pH Monitoring and Energy Harvesting

Wuliang Sun 1,2, Junhui Dong 1,*, Wenbo Li 3, Xiaobo Gao 4,5, Jun Liu 1 and Ding Nan 2,*

1 School of Materials Science and Engineering, Inner Mongolia University of Technology, Hohhot 010051, China
2 College of Chemistry and Chemical Engineering, Inner Mongolia University, Hohhot 010021, China
3 College of Food Science and Engineering, Inner Mongolia Agricultural University, Hohhot 010018, China
4 Institute of Applied Nanotechnology, Jiaxing 314031, China
5 Beijing Institute of Nanoenergy and Nanosystems, Chinese Academy of Sciences, Beijing 101400, China
* Correspondence: jhdong@imut.edu.cn (J.D.); nd@imu.edu.cn (D.N.)

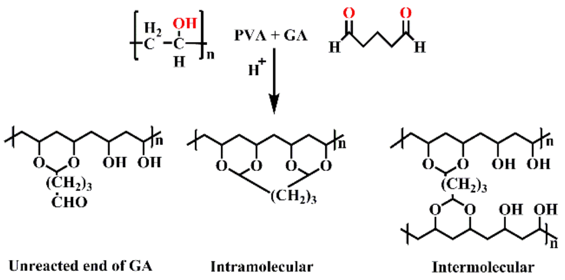

Figure S1. Chemical structure during the fiber film crosslinking reaction.

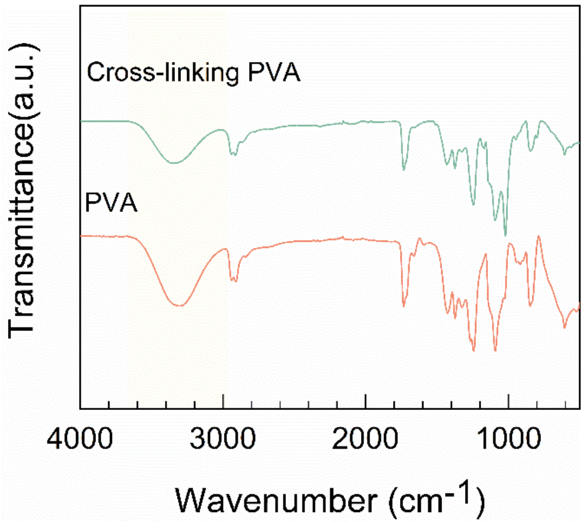

Figure S2. FT-IR spectra of different nanofiber membranes.

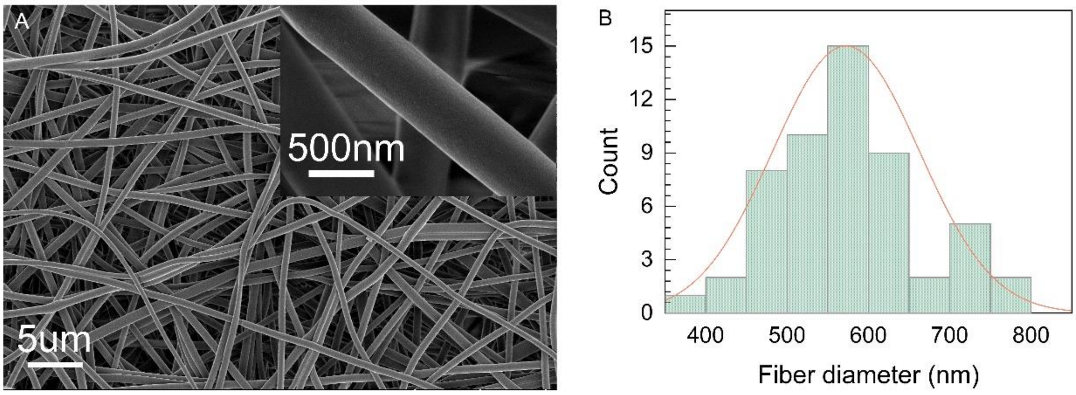

Figure S3. A)SEM image of pure PVA fiber film. B) Fiber diameter distribution of pure PVA fiber film.

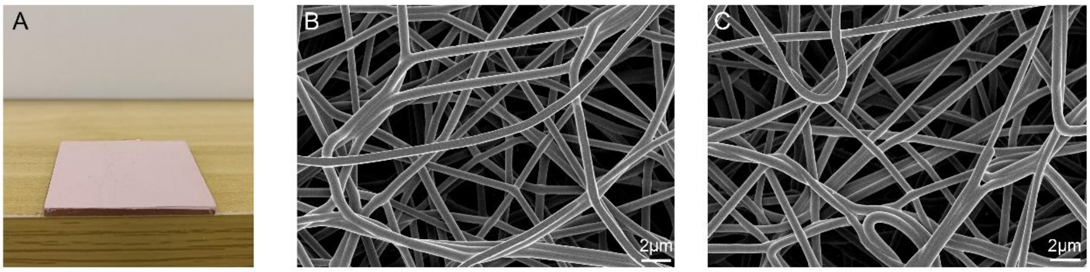

Figure S4.A) Photographs of PVA@Ac fiber surfaces following the durability experiments. B) SEM images of PVA@Ac fibers before the durability experiments. C) SEM images of PVA@Ac fibers after durability testing.

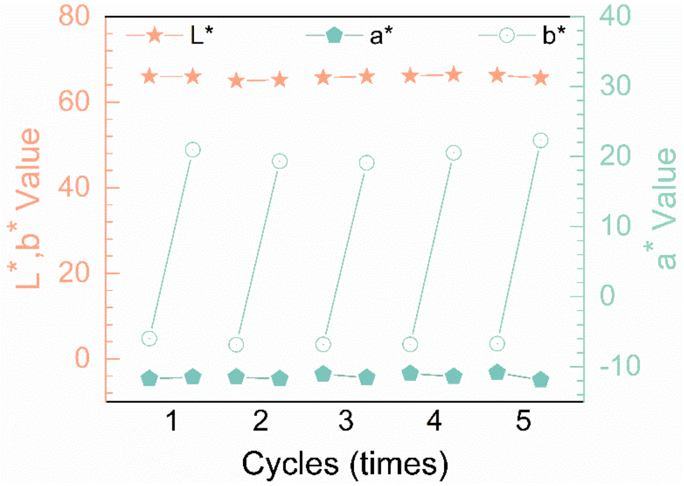

Figure S5. The Reusability of pH-TENG.

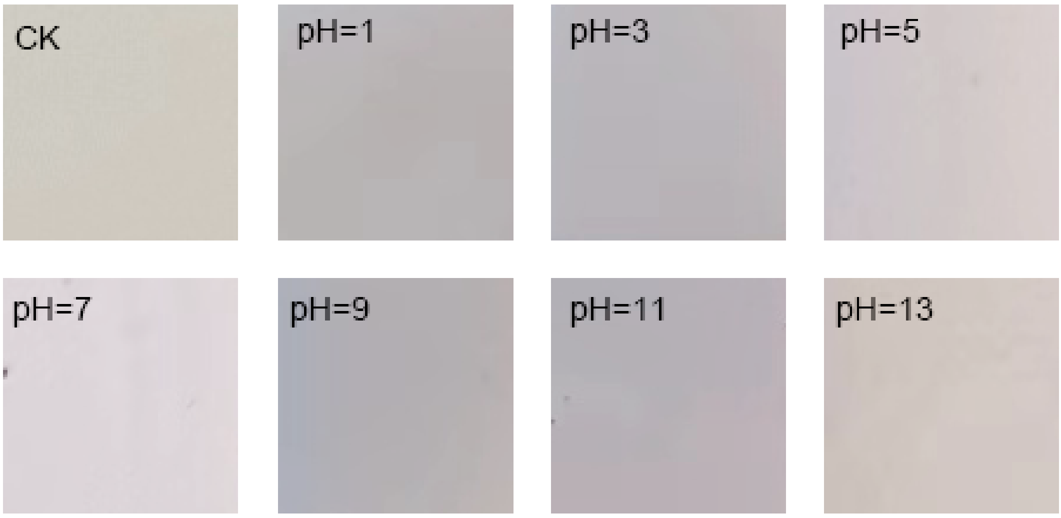

Figure S6. The color variations of pure PVA under different pH values.

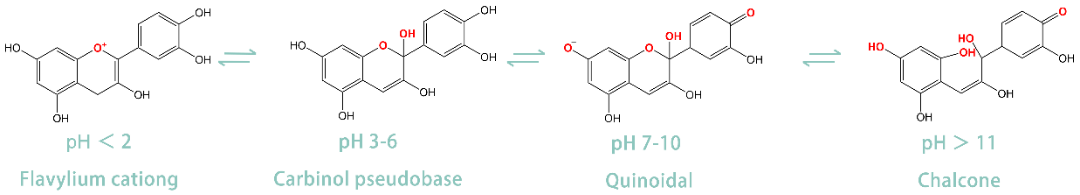

Figure S7. Structural transformations of anthocyanin in response to pH variations.
